# Segmentation of Striatal Brain Structures from High Resolution PET Images

**DOI:** 10.1155/2009/156234

**Published:** 2009-11-04

**Authors:** Ricardo J. P. C. Farinha, Ulla Ruotsalainen, Jussi Hirvonen, Lauri Tuominen, Jarmo Hietala, José M. Fonseca, Jussi Tohka

**Affiliations:** ^1^Department of Signal Processing, Tampere University of Technology, 33101 Tampere, Finland; ^2^Department of Electrical Engineering, Faculty of Science and Technology, New University of Lisbon, 2829-516 Caparica, Portugal; ^3^Ramboll Finland Oy, 02241 Espoo, Finland; ^4^Department of Psychiatry, University of Turku, 20700 Turku, Finland; ^5^Turku PET Center, Neuropsychiatric Imaging, Turku University Central Hospital, 20520 Turku, Finland

## Abstract

We propose and evaluate an automatic segmentation method for extracting striatal brain structures (caudate, putamen, and ventral striatum) from parametric ^11^C-raclopride positron emission tomography (PET) brain images. We focus on the images acquired using a novel brain dedicated high-resolution (HRRT) PET scanner. The segmentation method first extracts the striatum using a deformable surface model and then divides the striatum into its substructures based on a graph partitioning algorithm. The weighted kernel *k*-means algorithm is used to partition the graph describing the voxel affinities within the striatum into the desired number of clusters. The method was experimentally validated with synthetic and real image data. The experiments showed that our method was able to automatically extract caudate, ventral striatum, and putamen from the images. Moreover, the putamen could be subdivided into anterior and posterior parts. An automatic method for the extraction of striatal structures from high-resolution PET images allows for inexpensive and reproducible extraction of the quantitative information from these images necessary in brain research and drug development.

## 1. Introduction

**Figure 1:**
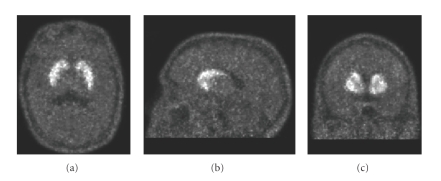
An example of one ^11^C-raclopride PET image of a human head: (a) transaxial, (b) sagittal, (c) coronal views.

POSITRON emission tomography (PET) is a widely used functional imaging technology. PET imaging allows measuring physiological processes in human's in vivo and computing three dimensional (3D) images quantifying the parameters of these processes. This functional information enhances the understanding of biochemical basis of normal and abnormal bodily functions [1]. The type of the functional information provided by the PET images depends on the applied radiopharmaceutical that is injected into the subject. To create quantitative 3D images of the physiological parameter of interest—so called parametric images—one needs to study the dynamics of the concentration of the applied radiopharmaceutical at each voxel and relate these to the input function. The input function is the concentration of the radiopharmaceutical in either arterial plasma or in a specific brain region known as the reference region [2]. In this study, raclopride labeled with ^11^C is used as the radiopharmaceutical. ^11^C-Raclopride is a selective antagonist and binds reversibly to dopamine D2 receptors. The parameter of interest in this case is the binding potential (*BP*) of D2 dopamine receptors which reflects the receptor density. The striatum contains the highest density of D2 receptors in the brain. In this work, we propose an automatic method to segment striatal substructures in ^11^C-raclopride PET images. Here, we consider *BP* as the ratio of specific to free plus nonspecific binding in tissue, and refer to it this point forward as *BP*_ND_ [2]. An example of a parametric image representing the *BP*_ND_ of ^11^C-raclopride is shown in Figure 1.

Most image segmentation in clinical environments is currently done by manual slice editing, meaning that a human operator manually delineates the region of interest (ROI) on each 2D slice of the 3D image. This process has several disadvantages. For example, the results will be dependent on any subjective bias on part of the operator deducing the spatial shape of 3D brain structures by viewing the 2D slices. Thus, the final result might not be reproducible by another operator, hampering the comparability between individual raters. Second, the enormous amount of data that must be manually or semiautomatically extracted makes the manual segmentation process time-consuming and expensive.

We have earlier presented an automatic method to extract striatal structures, namely left and right caudate and putamen, from ^11^C-raclopride PET images [3]. This method has been shown to produce more reproducible segmentations than the traditional manual slice editing [3, 4]. So far, this method has not been applied to the images acquired using the newest generation of PET scanners, the High-Resolution Research Tomograph (HRRT), because a new, improved method is needed for the segmentation of these images.

There are two primary reasons for this need. First, the segmentation method presented in [3] relies on the computation of the eigenvalues and eigenvectors of an image derived affinity matrix. However, when the image size increases, eigenvalue computation becomes computationally prohibitive especially regarding the memory requirements. The images acquired using a high-resolution PET scanner (ECAT HRRT, Siemens Medical Solutions, Knoxville, Tenn, USA) are of a size that exceeds the limit of making the eigenvalue computations impractical. The reconstructed dynamic images have approximately 800 Mbytes of data and the size of the affinity matrix is approximately 16000 × 16000. Second, the method used in [3] was designed to extract only caudate and putamen, but, with the resolution enhancement offered by HRRT, more substructures can be separated.

Accurate segmentation of the striatum from high-resolution PET images is of great interest because the functional anatomy of this structure is far more complex than what can be captured by previous generation PET scanners with lower spatial resolution. Indeed, the striatum can be divided based on topographically organized anatomical connections with cerebellar cortex into limbic (nucleus accumbens, and ventral parts of caudate and precommissural putamen), associative (most of the head of caudate and ventral parts of precommissural putamen), and sensorimotor (postcommissural putamen) divisions [5, 6]. This anatomical framework may have implications for the regional distribution of D2 receptors within the striatum. Therefore, in the present work, we introduce an automatic technique that is able to extract multiple striatal structures from parametric ^11^C-raclopride *BP*_ND_ images generated with HRRT PET.

The segmentation problem is divided in three parts, the extraction of the left and right striatum, the creation of a striatal image graph, and partitioning the striatal image graph into a predefined number of segments corresponding to striatal substructures. The extraction of the striatum is performed by a global optimization based deformable surface model. Then, a weighted striatal image graph, whose nodes correspond to striatal voxels, is constructed. The weights of the edges in the graph are computed based on the spatial locations and intensity values of voxels. The image graph can be represented by a matrix of the edge weights called the affinity matrix. Once the affinity matrix is constructed, we apply a graph partitioning algorithm to find the optimal partitioning. Spectral methods, such as normalized cuts algorithm applied in [3], are a widely used choice for partitioning. However, spectral clustering methods rely on the computation of the eigenvalues and eigenvectors of the affinity matrix to partition the graph. In cases where the large size of affinity matrix makes the eigenvector computation prohibitive, spectral methods will fail. The HRRT data used in this study produced large affinity matrix, rendering the spectral methods impractical. Therefore, our implementation of a graph partitioning method has to dwell with this data size problem. In [7] it was shown that the weighted kernel *k*-means clustering criterion with particular selections of kernel and weights is equivalent to the multiway normalized cuts criterion. The weighted kernel *k*-means criterion is a generalization of the well known *k*-means criterion. It can be locally optimized using a simple and fast algorithm similar to *k*-means clustering algorithm. This equivalence provides a principled way to avoid the eigenvalue computations to solve normalized cuts type graph partitioning criteria.

## 2. Methods

**Figure 2:**
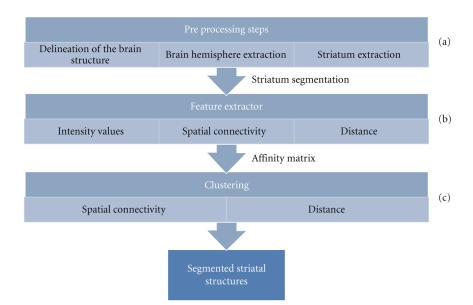
Steps of the segmentation of caudate, putamen, and ventral striatum: (a) preprocessing steps, (b) affinity matrix construction method, (c) clustering process.

Our method for the segmentation of striatal structures from HRRT PET images proceeds in a top-down manner. The automatic method consists of a series of consecutive steps shown in the hierarchy graph in Figure 2.

The preprocessing step consists of the extraction of the striatum, from HRRT PET *BP*_ND_ images, see Figure 2(a). This extraction method is the same as the one used in the earlier method [3] except for slight tuning of the initial parameters.

Once the striatum has been extracted an affinity matrix containing the similarity values between all pairs of striatal voxels is constructed using voxel coordinates and intensities as features, see Figure 2(b). We call this step feature extraction and it is described in Section 2.2.

Finally, the clustering algorithm segments the striatum into components corresponding to caudate, ventral striatum and anterior and posterior putamen, see Figure 2(c). The clustering process is treated as a graph partitioning problem, where the affinity matrix defines to the graph to be partitioned. The clustering algorithm is described in Section 2.3.

### 2.1. Preprocessing

Before we can proceed with the striatum segmentation, we need to segment left and right striatum from the background. This preprocessing can be subdivided in three steps: extraction of the brain surface using a deformable surface model [8], segmentation of the brain image in right and left brain hemispheres based on the extracted brain surface [8], and finally extraction of the striatum from each hemisphere [3]. The previous method [3] was found to be applicable for also HRRT-PET images with a minor parameter tuning and we refer to [3] for more detailed information on these methods.

### 2.2. Feature Extractor

The feature extractor aims to construct an affinity matrix using features extracted from the analyzed image. As we have already mentioned, the affinity matrix can be understood as a graph and vice versa. This graph contains the similarity values between all pairs of voxels belonging to the striatum. The image features used in our method are coordinates of voxel centers and voxel intensity values, that is, *BP*_ND_ values. Let *D* denote the set of voxels within left or right striatum and let *I*(*i*) be the intensity value of the voxel *i* = (*i*_*x*_, *i*_*y*_, *i*_*z*_) belonging to *D*. We define the similarity *A*(*i*, *j*)′ between voxels *i* and *j* as

(1) $${A\left(i,j\right)}^{\prime }=\{\begin{array}{cc} \exp\;\left(\frac{-2\left|I\left(i\right)-I\left(j\right)\right|}{{\max\;}_{k\in D}I\left(k\right)}\right) & \text{if }i\text{ is connected to}\;j\\ 0 & \text{otherwise}. \end{array}$$

The voxel *i* is said to be connected to the voxel *j* if *j* is included in the neighborhood of *i*. Voxels *i* and *j* are neighbors if the Euclidean distance between their centers is less than given constant. This constant should be selected to reflect the noise level and spatial resolution of the images to be segmented: with noisy images the constant needs to be lower than with less noisy images. Assuming that *I*(*i*) > 0 for all *i*, *A*(*i*, *j*) takes values between exp(−2) and 1 if the voxels *i* and *j* are connected. We still enhance the boundaries between the different structures by thresholding the affinity matrix. The final thresholded *A*(*i*, *j*) affinity matrix is defined as

(2) $$A\left(i,j\right)=\{\begin{array}{cc} {A\left(i,j\right)}^{\prime } & {A\left(i,j\right)}^{\prime }>PT\\ 0 & \text{otherwise.} \end{array}$$

In (2), *A*(*i*, *j*) corresponds to the final affinity value between voxels *i* and *j*, while *A*(*i*, *j*)′ is computed by (1). This second thresholding removes connections between any two voxels with *A*(*i*, *j*)′ inferior to a chosen threshold. This threshold is defined in (2) as Percentage Threshold (*PT*). The values of *PT* can vary from 0 to 1. In this work, we set *PT* = 0.5 based on experimental data.

### 2.3. Clustering Process

We partition the graph represented by the affinity matrix *A* into subgraphs corresponding the caudate, anterior and posterior putamen, ventral striatum, and background using weighted kernel *k*-means algorithm.

As already mentioned, the weighted kernel *k*-means algorithm is a generalization of the well known *k*-means algorithm. The weighted kernel *k*-means criterion function is [7]

(3) $$\;d\left({\left\{\pi_{i}\right\}}_{i=1}^{k}\right)=\overset{k}{\underset{i=1}{\sum }}\;\underset{a\in \pi_{i}}{\sum }W\left(a\right){\left\|\phi \left(a\right)-m_{i}\right\|}^{2}.$$

In (3), *π*_*i*_ denotes *i*th cluster with the cluster centre *m*_*i*_ = ∑_*b*∈*π*_*i*__ *W*(*b*)*ϕ*(*b*)/∑_*b*∈*π*_*i*__ *W*(*b*) and *W*(*b*) is the weight assigned to the voxel *b*. These weight values are computed based on the affinity matrix (2) as we will show in what follows. The nonlinear function *ϕ* maps the original (input) space to a higher-dimensional feature space. This mapping allows extracting the clusters that are not linearly separable in the input space. This mapping is also computed based on the affinity matrix.

Let *W* be a diagonal matrix of the weights *W*(*a*) for all voxels in *D* and *W*_*j*_ be a diagonal matrix of weights in the cluster *π*_*j*_. Further, collect all the inner products *ϕ*(*a*) · *ϕ*(*b*), *a*, *b* ∈ *D* into the kernel matrix *K*. The weighted kernel *k*-means criterion can be locally optimized by Algorithm 1.

The weighted form of the kernel *k*-means is mathematically equivalent to a general weighted graph clustering [7]. Such equivalence implies that the weighted kernel *k*-means algorithm can be used to locally optimize a number of graph clustering objectives such as normalized cuts. The equivalence of (3) to the normalized cuts criterion is obtained by selecting

(4) $$\begin{array}{c} W\left(i,i\right)=\underset{j}{\sum }A\left(i,j\right),\\ K=W^{-1}AW^{-1}, \end{array}$$

where *A* is the affinity matrix defined in (2).

 Although the weighted kernel *k*-means algorithm allows for segmenting a large number of striatal voxels into the desired substructures of interest, it has some subtleties. Namely, the final result depends on the desired number of clusters and the initial clustering. These parameters have to be carefully tuned to provide the most accurate result. We examined several different choices for these parameters and found an initial configuration that was both reliable and accurate in this application. Particularly, the initial clustering was formed by dividing the striatal domain *D* into *k* = 4 components of the equal size based on a lexicographical ordering. Voxels in the striatum were lexicographically ordered first scanning the domain from left to right direction, then from anterior to posterior direction, and finally from inferior to superior direction. Technically, for the image with dimensions *X* × *Y* × *Z*, each voxel *i* = (*i*_*x*_, *i*_*y*_, *i*_*z*_) in *D* was assigned a score *s*(*i*) = *i*_*x*_ + *Xi*_*y*_ + *XYi*_*z*_, where *i*_*x*_, *i*_*y*_, and *i*_*z*_ are coordinates of *i* in the left-right, anterior-posterior, and inferior-superior axes, respectively. The voxels were ordered based on these scores: the voxel *i* was initially assigned to the cluster *π*_*c*_ if (*c* − 1)|*D*|/4 < *r*(*i*) ≤ *c*|*D*|/4, where *r*(*i*) is the rank of *s*(*i*) among the voxels in *D*. No differences to the final results were observed if instead of scanning from left to right we would scan from lateral to medial in the case of the right striatum. Note that the initialization process is deterministic, that is, there is no random component in our algorithm. This is important for the current application because it ensures the reproducibility of the automatic segmentations.

## 3. Experiments

### 3.1. Phantom Study

We performed experiments with simulated HRRT PET phantoms to evaluate the automatic segmentation method. For this, we generated dynamic phantoms corresponding ^11^C-raclopride brain images. The construction of the phantoms required a model of anatomy (a labeled 3D brain volume) and a model of physiology (Time Activity Curves (TACs) for each brain region).

The anatomical model was derived from a manually segmented T1-weighted brain MR image (the Jacob phantom by Montreal Neurological Institute [9]). We reduced the number of brain structures represented in the Jacob phantom to 9 distinct anatomical structures for our anatomical model. The structures in the anatomical model were anterior (dorsal) and posterior putamen, ventral striatum (including only nucleus accumbens in our model), caudate (dorsal), white and gray matter, cerebellum, sinuses, scalp, and skull. The putaminal subdivision was defined according to previously published criteria [10].

The physiological model consisted of a set of TACs, each one describing the dynamic behavior of the radiopharmaceutical in a different anatomical structure. The TAC's for the individual structures were taken from [11]. In [11], the model of physiology did not include separate TACs for posterior/ anterior putamen or ventral striatum. Therefore, an estimation of those TAC's had to be done in order to test our method properly. The TAC for the anterior putamen was obtained by multiplying the TAC of the posterior putamen by a constant (1.05). Similarly, the TAC for the ventral striatum was obtained by multiplying the caudate TAC by a constant (0.9). These are reasonable approximations based on our experience with these data. We additionally generated images using a set of different constant values. Different constants led to different *BP*_ND_ values in the structures, and for us, increased or decreased contrast between the structures. However, slightly altered contrasts caused only minor changes to the segmentation results and therefore we only report quantitative results with phantoms constructed using the physiological model specified above.

**Figure 3:**
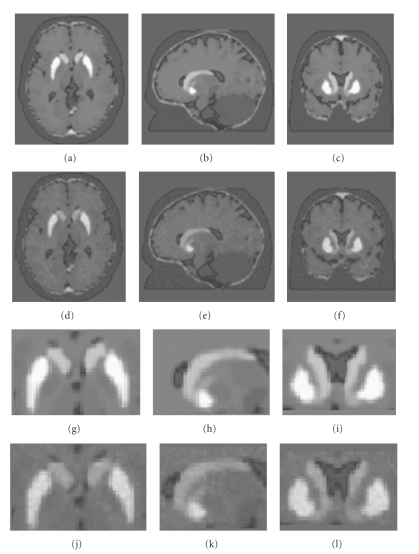
HRRT phantom. (a), (b), and (c) represent the transaxial, sagittal, and coronal views of the HRRT phantom with the ideal noise level, while (d), (e), and (f) represent to the same phantom with the more realistic noise level. Figures 3(g) to 3(l) are the zoomed (400%) to the striata in the images presented in (a) to (f).

The noiseless dynamic PET phantom was generated by assigning each voxel and each time frame to a count value based on the anatomical model used and the time activity curves (TACs) of the radiopharmaceutical. The dimensions for the phantom were 256 × 256 × 200 with 26 time frames. The voxel size was 1.22 mm × 1.22 mm × 1.22 mm. The phantom was convolved with a 2.5 mm^3^ Gaussian kernel to model the resolution of the ECAT HRRT scanner [12]. After generating radioactive decay effects, noise was added by drawing voxelwise counts from the Poisson distribution with the mean as the count value in the noiseless convolved phantom. This ideal level of noise is much lower than the one encountered in reality due to the effects of scatter, attenuation, randoms, and detector dead time. In addition to the phantom with ideal noise level, we generated a phantom with a more realistic noise level. For this we multiplied the voxel values by an estimated survival probability of 0.04 before generating the noise. The survival probability models the probability that a photon pair resulting from positron-electron annihilation is detected by the scanner. Our rough estimate of the survival probability was based on the approximation that 80% of the true counts are lost due to attenuation and other 80% are lost due to detector dead-time. Other factors affecting the noise level were ignored in this estimate. The performance evaluations of HRRT PET scanners are available [12], but linking these performance characteristics to image noise estimates is challenging [13], and thus we settled for this rough noise level estimate. The images were decay corrected, multiplied by the inverse of the survival probability and the units were converted to nCi/ml. Finally, the *BP*_ND_ image was computed using the simplified reference region model using the cerebellum as the reference region [14]. Examples of the phantom with ideal and realistic noise levels can be seen in Figure 3.

With the phantom images, it is possible to quantitatively evaluate our method because we know the ground truth *BP*_ND_ values for each structure. Because the main interest was in evaluating the accuracy of the extracted regional *BP*_ND_ values, we limit the discussion on the evaluation of the accuracy of structure-wise *BP*_ND_ values computed based on automatic segmentation. The structure-wise *BP*_ND_ values were computed by averaging the voxel-wise *BP*_ND_ values in a parametric image for a given structure (left and right caudate, ventral striatum, and anterior and posterior putamen). The performance of our method was measured using the normalized absolute similarity (NAS), which is defined:

(5) $$\text{ NAS}=1-\left(2\frac{\left|BP_{a}-BP_{b}\right|}{\left({BP}_{a}+{BP}_{b}\right)}\right).$$

In (5), *BP*_*a*_ and *BP*_*b*_ represent, respectively, the average *BP*_ND_ values computed based on the automatic segmentation and the ground truth *BP*_ND_ value computed based on the physiological ground truth TAC. The NAS values range from −1 to 1 assuming that *BP*_ND_ values are positive. This and closely related performance measures are widely applied to assess test-retest variability within PET imaging (e.g., [3, 4, 15]) which simplifies its interpretation relative to other studies.

We also compared the automatically segmented volumes to the anatomical ground-truth volume. The applied figure of merit was positive predictive value (PPV) introduced in our earlier work [3] to estimate the quality of PET image segmentations. PPV was computed for every structure separately. We define *true positives (TPs) *as voxels which truly belong to a certain brain structure and are also labeled as belonging to that same brain structure in an automatic segmentation. *False positives (FPs*) are voxels which do not truly belong to a brain structure in the anatomical ground-truth but are labeled as belonging to it in an automatic segmentation. PPV is now defined as

(6) $$\frac{\left|TP\right|}{\left|TP\right|+\left|FP\right|},$$

where |*TP*| (|*FP*|) is the number of TPs (FPs). PPV is used as the figure of merit here, because, as argued in [3], it is important that an extracted brain structure contains only voxels truly belonging to that brain structure and it is not that important whether or not we detect all the voxels belonging to that brain structure.

### 3.2. PET Studies with Healthy Volunteers

We compared our automatic segmentation method to manual ROI segmentation. This comparison was done using ^11^C-raclopride *BP*_ND_ high-resolution PET images. The PET scans were provided by Turku PET Center and were acquired from four right-handed, nonsmoking healthy male volunteers. This study was approved by the Joint Ethical Committee of the University of Turku and Turku University Central Hospital, and was conducted according to the Declaration of Helsinki. All subjects gave ethical committee-approved written informed consents. All subjects were free of any somatic or psychiatric illness, illicit drug abuse, and alcoholism, as confirmed by blood and urine tests, somatic assessment, and a structured clinical interview for DSM-IV Axis I disorders conducted by a psychiatrist. The age, height, and weight of the subjects were 27.5 ± 7.74; (20.3 to 33.1) years, 175 ± 1 (174 to 176) cm, and 76.1 ± 5.16 (70.0 to 82.5) kg, respectively (mean, s.d.; range). All subjects underwent T1-weighted magnetic resonance imaging at 1.5T to rule out structural brain abnormalities.

The radioligand ^11^C-raclopride was prepared as previously described [15]. PET imaging was performed by a brain-dedicated ECAT HRRT, Siemens Medical Solutions, Knoxville, Tnee, USA) [12]. In the acquired measurements, a molded thermoplastic mask was used as a head fixation to reduce the noise due to head movements. The right antecubital vein was cannulated and ^11^C-raclopride was administered as an intravenous rapid bolus flushed with saline. Injected dose and the specific radioactivity were 443.25 ± 104.69 MBq and 185.7 ± 218.4 MBq/nmol, respectively (mean ± S.D.). The PET ^11^C-raclopride scans were acquired in list mode and histogrammed by axial compression of span 9. The ^11^C-raclopride uptake was measured for fifty one minutes after injection and the frame sequence consisted of three frames of sixty seconds, four frames of one hundred and eighty seconds, and six frames of three hundred and sixty seconds. The image size of each scan was 200 × 200 × 150 slices and they had an isotropic voxel dimension of 1.22 × 1.22 × 1.22 mm^3^. The raw data was reconstructed with a speed-optimized version of OP-OSEM-3D (Ordinary Poisson-OSEM in full 3D) [16], with sixteen subsets and eight full iterations [17]. The frame-images of each dynamic PET image were first realigned to correct for head movement during the scan. Integral image (summed over time) was calculated from the realigned PET-image and the MRI was coregistered to this PET-image. All realignment and coregistration procedures were performed using Statistical Parametric Mapping software version 2 (SPM2, Wellcome Department of Cognitive Neurology, University College London, UK). The parametric *BP*_ND_ images were created from the dynamic images using the simplified reference tissue model with the cerebellar cortex as the reference region [14].

Regions of interest (ROIs) were manually drawn to coregistered transaxial MRI using Imadeus software (version 1.2., Forima Inc., Turku, Finland). ROIs onto dorsal caudate, dorsal putamen, ventral striatum, thalamus, and cerebellum were defined as previously described [16]. Simplified reference tissue model with the cerebellar cortex as the reference region [14] was applied to TAC data to obtain the regional *BP*_ND_ values.

Average NAS (ANAS) over the four subjects was used to measure the similarity of the *BP*_ND_ values based on the automatic segmentation and the manual segmentation. In this case the *BP*_*a*_ and *BP*_*b*_ in (5) represent, respectively, the *BP*_ND_ values computed based on the automatic segmentation and manual segmentation. In this case, however, it cannot be claimed that the *BP*_ND_ values based on the manual segmentation would be correct although they can be used as benchmarks.

## 4. Results

### 4.1. Phantom Study

Table 1 presents the *BP*_ND_ and NAS values from automatic segmentations of caudate, ventral striatum, putamen, anterior and posterior putamen. These results were obtained when applying the automatic method to ideal and realistic noise phantoms. The voxel connectivity threshold in (1) was set to 10 with the phantom images.

Table 1 shows that the ideal phantom NAS results were between 92.3% and 99.9%, while the phantom with a realistic noise level had NAS values between 94.9% and 99.9%. In a test-retest setting, where the same subject is studied twice with a short interval between the scans, the similarity of *BP*_ND_ estimations is typically in the range of 90%–95% [3, 18]. Therefore, the automatic segmentations provided accurate *BP*_ND_ values in the phantom experiments, in that the deviation from the ground truth was less than the typical test-retest variability. Moreover, the NAS values presented in Table 1 also indicate that the increase of noise level did not affect the NAS values, suggesting that the automatic method was resistant to noise. The PPV values were high for caudate and anterior putamen indicating that the functionally driven segmentations were also in an excellent agreement with the anatomy with respect to these structures. The PPV values for posterior putamen were lower resulting from the posterior putamen in automatic segmentation containing many partial volume voxels between posterior and anterior putamen, and also, between anterior putamen and surrounding white matter tissue. This was expected since the *BP*_ND_ values of the partial volume voxels between anterior putamen and surrounding white matter were similar to *BP*_ND_ values of voxels in posterior putamen. The PPV values for ventral striatum were low reflecting the mixing of ventral striatum and caudate in automatically segmented volumes. This was due to small size of the structure, 954 voxels when both left and right hemispheres were considered, majority of which were partial volume voxels (922 voxels had a neighboring voxels belonging to another structure) in the anatomical ground truth. However, while the segmentation of ventral striatum was anatomically incorrect, the *BP*_ND_ values obtained based on it were accurate. This was explained by the fact that the majority of the voxels incorrectly labeled as ventral striatum were partial volume voxels near the boundary of caudate and ventral striatum. Thus, we do not consider the low PPV values of ventral striatum particularly worrisome. Note also that we have taken nucleus accumbens to represent ventral striatum while ventral striatum also encompasses some adjacent areas.

### 4.2. PET Studies with Healthy Volunteers

Table 2 presents the average *BP*_ND_ values obtained based on the automatic and the manual segmentation of the HRRT images of four healthy subjects. The average *BP*_ND_ values attained when applying manual segmentation to the real test data were 3.54 ± 0.53 for caudate, 4.26 ± 0.61 for putamen, and 3.42 ± 0.59 for ventral striatum. Note that the *BP*_ND_ values are average values across hemispheres and subjects. The average *BP*_ND_ values obtained with the automatic method were 2.97 ± 0.59 for caudate, 3.85 ± 0.64 for putamen, and 3.24 ± 0.57 for ventral striatum.

As can be seen from Table 2, the *BP*_ND_ values for the automatic and manual segmentations were similar for all structures and ANAS varied from 78% to 91%. These ANAS values indicate that the variations in regional *BP*_ND_ due to ROI extraction method were small compared to variations due to other choices in the image processing (e.g., the reconstruction and the parametric image generation method). The ANAS values were smallest in caudate. By visually inspecting the automatic segmentation results, it could be seen that the caudate cluster did not only include caudate, but also some white matter. This was probably the reason for the lower *BP*_ND_, and thereby, lowers NAS values for the caudate.

**Figure 4:**
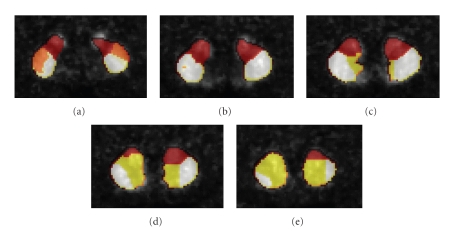
Segmentation of the right and left striatum using the automatic method. Coronal (a) slice 115, (b) slice 112, (c) slice 109, (d) slice 106, and (e) slice 103 magnified to show only the striatum. The orange and white clusters correspond, respectively, to the posterior and the anterior putamen, the red cluster corresponds to the caudate and, the yellow cluster corresponds to the ventral striatum. It can be seen that ventral striatum contains also a part of the caudate head.

Figure 4 shows the result of applying the automatic segmentation method to both left and right striatum from one of the HRRT PET scans. The results are from slices 115 (a), 112 (b), 109 (c), 106 (d), and 103 (e) from the coronal view. The segmentation results were appropriate, namely, the algorithm found four clusters corresponding to anterior putamen, posterior putamen, ventral striatum, and caudate. In Figure 4 the orange and white clusters correspond, respectively, to the posterior and the anterior putamen, the red cluster corresponds to the caudate and, the yellow cluster corresponds to the ventral striatum and a part of the caudate head.

**Figure 5:**
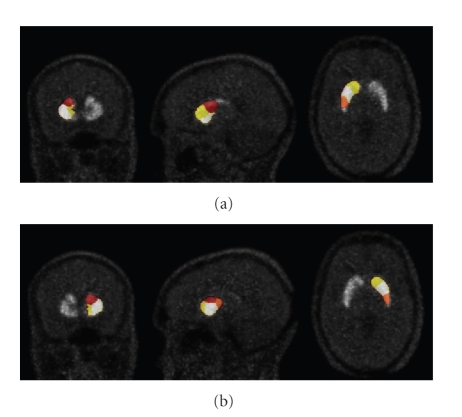
Segmentation result using the automatic method: (a) left striatum and (b) right striatum. In both images (a), (b), the orange and white clusters correspond, respectively, to the posterior and the anterior putamen, the red cluster corresponds to the caudate and, the yellow cluster corresponds to the ventral striatum and a part of the caudate head.

The results presented in Figure 5(a) are from slices 110, 115, and 120, from coronal, sagittal, and transverse views, while in Figure 5(b) they are from slice 110, 145, and 120. The images presented in Figures 4 and 5 are taken from the same clustering result.

Interestingly, the automatic method was able to divide the putamen into two substructures. A slight difference between *BP*_ND_ values within anterior and posterior putamen was observed in this study. Table 3 shows the *BP*_ND_ values from anterior and posterior putamen from all the four HRRT PET images. Unfortunately, the manual segmentation of these putaminal subdivisions was not available and we cannot compare the automatic *BP*_ND_ values with the manual ones. However, by inspecting Table 2, it can be seen that the similarity between the putamen *BP*_ND_ values obtained with the help of automatic and manual segmentations was approximately 90%. This indicates that the whole putamen was segmented very similarly with automatic and manual methods what reassures the correctness of the automatic segmentation. Moreover, when the method was applied to the phantoms, where the *BP*_ND_ values from those subdivisions were known, the algorithm accurately segmented both subdivisions (Table 1). These two facts allow us to assume that this separation was correct.

In Table 3, *BP*_ND_ values from Image1 are lower than for other images. The same was visible from the whole putamen *BP*_ND_ values based on the manual segmentation: left and right putamen average *BP*_ND_ was 3.41 for Image1 while it was greater or equal than 4.27 for other images. In the visual inspection, the *BP*_ND_ Image 1 appeared considerably noisier compared to the other images featured in this study, and especially, posterior putamen had also lower *BP*_ND_ values compared to the other images. The reasons for this might include the low injected dose (292 MBq), imaging artifacts (e.g., subject movements), and individual variability.

The final segmentation results were found to be highly dependent on the initial clustering guesses when applying the kernel weighted *k*-means algorithm to subdivide the striatum. The best segmentation results were obtained when the initial clusters were separated into four clusters each with the same size. A random initialization resulted usually in a single cluster consisting of the entire striatum. The neighborhood parameter in (1) needed also to be tuned to achieve the best segmentation. A voxel connectivity threshold of 4 voxel-sides achieved the best results. This value was different from the phantom experiments due to more complex noise patterns with real data. It was also noted that the method always resulted in four clusters even if the number of the initial clusters was greater than four. In other words, starting with more than four clusters resulted in empty clusters.

## 5. Discussion

A new automatic method was developed to extract striatal brain structures, namely ventral striatum, caudate, anterior putamen and posterior putamen, from parametric HRRT ^11^C-raclopride PET images. This anatomical subdivision of the striatum has relevant functional implications. An automatic method for the extraction of these structures from high-resolution PET images allows for inexpensive and reproducible extraction of the quantitative information from these images necessary in brain research and drug development. The method developed in this study proved to be accurate and reproducible, and was found to provide more information about the distribution of D2 receptors within the striatum than manual segmentation. We evaluated it using both images from healthy volunteers and simulated images (phantoms).

The method is based on the extraction of the striatum using deformable surfaces. The extracted striatum is then sub-divided into four clusters corresponding to ventral striatum, caudate and anterior and posterior putamen using the weighted kernel *k*-means algorithm. In this clustering, we take into account both the intensity value, in this case representing binding potential, of the voxel as well as its spatial location with respect to the other striatal voxels. We chose to use the weighted kernel *k*-means criterion for the clustering because of its recently established equivalence to the normalized cuts criterion. The weighted kernel *k*-means algorithm can be seen as a fast algorithm to optimize the multiway normalized cuts criterion. Earlier methods to optimize this criterion require a solution of eigenvectors of a large matrix, which could be computationally difficult. Indeed, the new algorithm, programmed in MATLAB, required only few minutes to segment the striatum.

 In the algorithm, a few parameter values needed to be set (see Sections 2.2 and 2.3). All the parameter values were set in the same way in all the experiments with a single exception of the connectivity threshold set differently between the phantom and the real data experiments. The reason for this was the obvious difference between the phantoms and real PET studies. The noise model in the phantom studies was highly simplified and it either ignored or simplified many aspects that affect the appearance of parametric *BP*_ND_ images in reality. Also, the spatial resolution of the simulated phantom images was higher than in the actual studies because the effects of the image reconstruction were ignored. Simulating more realistic computer generated PET images requires advanced computational methods (see, e.g., [19]) and these Monte Carlo simulators are not yet directly available for modeling HRRT-PET images. We do not think that the setting of the parameter values for the method would present a major hurdle to the automatic application of the method to a larger set of HRRT-PET images.

In the experiments with the simulated images, the numbers of voxels in the substructures in the automatic segmentations differed from the numbers of voxels in the anatomical ground truth. In the anterior putamen and caudate, the numbers of voxels in the automatic segmentation were lower than in the ground truth. This is a desired feature of the segmentation algorithm because otherwise partial volume effects (PVEs)—here referring to the combined effect of the tissue fraction effect and the point spread effect in the terminology of [20]—could compromise the quantitative accuracy of the regional *BP*_ND_ values (see discussions on this issue in [3, 4]). While the resolution of PET images acquired by the HRRT is superior to the resolution of PET images acquired by earlier PET scanners, the image resolution and PVEs still continue to be a limiting factors of the accuracy of the image segmentation. The numbers of voxels in the ventral striatum and posterior putamen were greater in the automatic segmentation than in the anatomical ground truth. We attribute also this to the PVEs although it was not a desired feature of the segmentation algorithm. In the case of ventral striatum, the majority of extra voxels were partial volume voxels near the boundary of caudate and ventral striatum, thus having lower *BP*_ND_ values than “pure” caudate voxels. Similarly, the majority of extra voxels assigned to posterior putamen were partial volume voxels between the anterior and posterior putamen. Hence, the effects of these segmentation errors on the accuracy of the regional *BP*_ND_ values were negligible as it visible in NAS values in Table 1.

Although the accuracy or regional *BP*_ND_ values—the computation of which was the reason behind designing the segmentation algorithm—were accurate it would be advantageous if the segmentations themselves were more accurate, especially with respect to ventral striatum. There are a number of research avenues one could follow while trying to improve the algorithm. The extraction of striatum could be improved by taking the PVEs better into account. This could be achieved by adopting a volume based segmentation strategy based on, for example, Markov Random Fields, instead of the present edge-based strategy. However, this is not straight-forward because the *BP*_ND_ values within the striatum are highly nonuniform due to *BP*_ND_ differences between striatal substructures, noise, and PVEs. Moreover, in the case of the HRRT scanner, the PVE is challenging to model due to gaps between adjacent flat panel detector heads. The segmentation of striatum could also include modeling of the PVE and neuroanatomical a priori knowledge could be better utilized either in the kernel weighted *k*-means criterion or in the initialization for the kernel weighted *k*-means algorithm.

The ability of the automated method to divide the putamen into anterior and posterior components was an originally unexpected result. This ability is truly interesting as the distribution of D2 dopamine binding within the putamen has been rarely studied. This is partly due to the fact that with the earlier generation PET scanners differences between anterior and posterior putamen are less evident due to low image resolution. Also, there are no generally accepted guidelines for dividing the putamen between anterior and posterior components manually; for instance, we previously employed a crude division half-way along the anterior-posterior axis of the putamen [10]. For this reason, we did not attempt putaminal subdivisions based on the manual segmentation a priori. Instead, we chose to evaluate the automatic segmentation results based on visual inspection, which indicated that the division was indeed highly reasonable. Moreover, in the studies with simulated images, the quantitative values obtained for the putamen subdivisions (anterior and posterior putamen) based on automatic segmentation were highly similar to the ground truth. The division to anterior and posterior putamen is consistent with a decreasing rostrocaudal gradient in dopamine D1/D2 receptor density in the human striatum [10], and may be physiologically relevant. For instance, D2 receptors are inhibitory on striatal output neurons, and differential expression of D2 receptor in functional subdivisions of the putamen may underlie differential dopaminergic modulation of associative versus motor functions [5, 6]. The fact that the direction of the contrast was not uniform across the subjects in this small sample (i.e., some had decreasing and some had increasing anterior-posterior gradients) might suggest inter-individual differences in preferential dopaminergic modulation of associate versus motor functions, but larger samples would be required to confirm this.

This method for automatic extraction of volumes could be further developed to segmentation of other types of PET images. Especially, the method is applicable to extract striatal substructures from PET images acquired using other radiopharmaceuticals than ^11^C-raclopride if the images share the characteristics of the ^11^C-raclopride images, that is, the image intensity is the highest in striatum. An example of such radiopharmaceutical is ^18^F-SPA-RQ used to study brain neurokinin NK1 receptors. The automatic segmentation of low resolution ^18^F-SPA-RQ PET images was studied in [4].

It would be conceptually straight-forward to modify the proposed method to work directly with 4-D PET images, where each voxel would be represented by a time activity curve—instead of 3D parametric images where each voxel is represented by a *BP*_ND_ value computed based on the time activity curve. Technically, this would entail replacing the term |*I*(*i*) − *I*(*j*)|/max_*k*∈*D*_ *I*(*k*) in (1) by an appropriate distance measure between two time activity curves. The distance measure could be, for example, the Euclidean distance between the vector representations of radioactivity measurements in different time intervals or a more sophisticated L_2_-norm between the time activity curves viewed as continuous valued functions in a suitable function space. However, the specification of the meaningful distance measure might prove troublesome in practice. In the current work, our interest was in the segmentation of striatum based on *BP*_ND_ values, and other information derivable from time activity curves was therefore not relevant for this application. We would not rule out the possibility to segment striatum without *BP*_ND_ computation as a preprocessing step, however, the development of such a method could be challenging in practice.

## Figures and Tables

**Algorithm 1:**
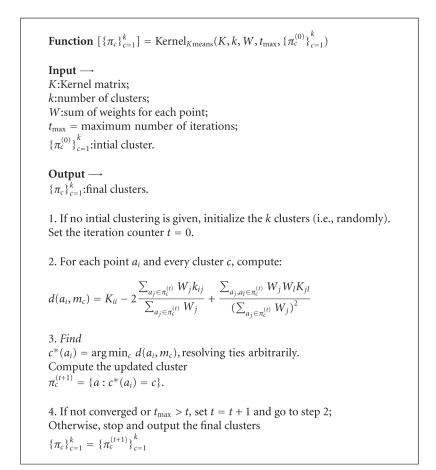
Graph partition algorithm (Weighted Kernel *k*-means). This algorithm assigns the voxel *a*_*i*_ to the cluster *π*_*c*_ if the cluster *π*_*c*_ minimizes the distance function from point 2 [7].

**Table 1:** *BP*_ND_ values, NAS values, the number of voxels in each brain structure, and PPV values computed based on the ideal phantom and the realistic noise phantom.

		Mean *BP*_ND_ value	NAS	# voxels	PPV
*Ideal phantom*					

Caudate					

Average	Automatic	1.614	0.9834	3672	0.8295
Average	Ground truth	1.641		6190	

Ventral striatum					

Average	Automatic	1.351	0.9817	2325	0.0714
Average	Ground truth	1.376		954	

Putamen					

Average	Automatic	2.698	0.9897	4874	0.9984
Average	Ground truth	2.726		6289	

Posterior Putamen					

Average	Automatic	2.524	0.9230	2639	0.4433
Average	groung truth	2.726		1929	

Anterior Putamen					

Average	Automatic	2.911	0.9993	2235	0.9817
Average	Ground truth	2.913		4360	

*Realistic noise phantom*					

Caudate					

Average	Automatic	1.655	0.9915	4340	0.7005
Average	Ground truth	1.641		6190	

Ventral striatum					

Average	Automatic	1.403	0.9806	2399	0.0354
Average	Ground truth	1.376		954	

Putamen					

Average	Automatic	2.854	0.9541	5063	0.9891
Average	Ground truth	2.726		6289	

Posterior Putamen					

Average	Automatic	2.591	0.9492	2271	0.5042
Average	Ground truth	2.726		1929	

Anterior Putamen					

Average	Automatic	2.804	0.9619	2792	0.9362
Average	Ground truth	2.913		4360	

**Table 2:** *BP*_ND_ and ANAS values obtained from the average of the HRRT PET data. These measurements allow the similarity analysis between the automatic and manual segmentations.

		mean ± *SD*	ANAS
Caudate			

Left	Manual	3.56 ± 0.46	0.78
Left	Automatic	2.86 ± 0.41	

Right	Manual	3.53 ± 0.59	0.84
Right	Automatic	3.07 ± 0.79	

Average	Manual	3.54 ± 0.53	0.81
Average	Automatic	2.97 ± 0.59	

Putamen			

Left	Manual	4.29 ± 0.63	0.89
Left	Automatic	3.86 ± 0.60	

Right	Manual	4.24 ± 0.61	0.90
Right	Automatic	3.84 ± 0.69	

Average	Manual	4.26 ± 0.61	0.90
Average	Automatic	3.85 ± 0.64	

Ventral striatum			

Left	Manual	3.41 ± 0.57	0.91
Left	Automatic	3.16 ± 0.57	

Right	Manual	3.43 ± 0.61	0.86
Right	Automatic	3.35 ± 0.71	

Average	Manual	3.42 ± 0.59	0.90
Average	Automatic	3.24 ± 0.57	

**Table 3:** *BP*_ND_ values from anterior and posterior putamen from all 4 HRRT PET scans.

		Binding potential values
Anterior putamen		

Image 1	Left	3.5810
	Right	3.2744

Image 2	Left	3.8194
	Right	3.7010

Image 3	Left	3.8194
	Right	3.5784

Image 4	Left	4.4652
	Right	4.7607

Posterior putamen		

Image 1	Left	2.5193
	Right	1.9575

Image 2	Left	4.3798
	Right	4.6810

Image 3	Left	4.3961
	Right	4.2066

Image 4	Left	4.5961
	Right	4.3590
